# dbVar structural variant cluster set for data analysis and variant comparison

**DOI:** 10.12688/f1000research.8290.2

**Published:** 2017-02-28

**Authors:** Lon Phan, Jeffrey Hsu, Le Quang Minh Tri, Michaela Willi, Tamer Mansour, Yan Kai, John Garner, John Lopez, Ben Busby

**Affiliations:** 1 National Center for Biotechnology Information, National Library of Medicine, National Institutes of Health, Bethesda, MD, USA; 2 Cleveland Clinic Lerner Research Institute, Cleveland, OH, USA; 3 Department of Biotechnology, Ho Chi Minh City International University, Ho Chi Minh, Vietnam; 4 Laboratory of Genetics and Physiology, National Institute of Diabetes, Digestive and Kidney Diseases, National Institutes of Health, Bethesda, MA, USA; 5 Division of Bioinformatics, Biocenter, Medical University Innsbruck, Innsbruck, Austria; 6 Lab for Data Intensive Biology, Department of Population Health and Reproduction, University of California, Davis, CA, USA; 7 Department of Clinical Pathology, University of Mansoura, Mansoura, Egypt; 8 Cancer Epigenetics Laboratory, Department of Anatomy and Regenerative Biology, The George Washington University, Washington, DC, USA; 9 Department of Physics, The George Washington University, Washington, DC, USA

**Keywords:** NCBI, dbVar, Structural Variation Cluster, GVF, Genomics, Open-Source, Genome Annotation, Education, Software

## Abstract

dbVar houses over 3 million submitted structural variants (SSV) from 120 human studies including copy number variations (CNV), insertions, deletions, inversions, translocations, and complex chromosomal rearrangements. Users can submit multiple SSVs to dbVAR  that are presumably identical, but were ascertained by different platforms and samples,  to calculate whether the variant is rare or common in the population and allow for cross validation. However, because SSV genomic location reporting can vary – including fuzzy locations where the start and/or end points are not precisely known – analysis, comparison, annotation, and reporting of SSVs across studies can be difficult. This project was initiated by the Structural Variant Comparison Group for the purpose of generating a non-redundant set of genomic regions defined by counts of concordance for all human SSVs placed on RefSeq assembly GRCh38 (RefSeq accession GCF_000001405.26). We intend that the availability of these regions, called structural variant clusters (SVCs), will facilitate the analysis, annotation, and exchange of SV data and allow for simplified display in genomic sequence viewers for improved variant interpretation. Sets of SVCs were generated by variant type for each of the 120 studies as well as for a combined set across all studies. Starting from 3.64 million SSVs, 2.5 million and 3.4 million non-redundant SVCs with count >=1 were generated by variant type for each study and across all studies, respectively. In addition, we have developed utilities for annotating, searching, and filtering SVC data in GVF format for computing summary statistics, exporting data for genomic viewers, and annotating the SVC using external data sources.

## Introduction

There is a growing body of evidence suggesting that genomic structural variants play an important role in the etiology of human disease and in determining individuals’ characteristics and phenotypes ^1, 2^. Structural variants are also important for understanding the evolution of species ^3^. dbVar is a database of large structural genomic variants that catalogs millions of records from both small and large studies and makes them freely available to the public ^4, 5^. The data are organized by submitted study, which makes for convenient comparisons between cases and controls. dbVar online search and browser tools make it easy to search and retrieve the data.

**Figure 1.**
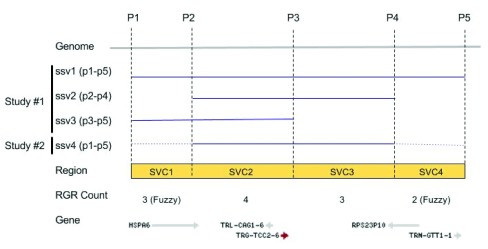
An alignment of variants ssv1-ssv3 (blue lines) with the genome (grey line) between positions P1 and P5. Reference genomic regions SVC1-SVC4 (yellow box) are demarcated by overlap and non-overlapping positions (P1-P2, P2-P3, etc.) between SSVs. The observed SVC counts and the genes are shown on the bottom.

It is difficult to annotate novel SVs or to compute summary data without a reference record or exemplar when multiple SSV choices are available in the same genomic region, and there has been no publicly available resource to date that combines variants from all studies for integration into a bioinformatic pipeline for search, analysis, and comparison. We created structural variant clusters (SVC) to overcome these problems. Structural variant clusters ( Figure 1) are smaller discrete genomic features that include counts of the features shared between SSVs. In regions with fuzziness between overlapping SSVs, SCVs allow the calculation of annotation and frequency by either consensus overlapping regions or by user-defined limits.

Additional benefits of having a defined set of SVCs include:

- improved data exchange, data mining, computation, and reporting;
- better searching and matching of genomic coordinates across studies;
- easier aggregation of annotations such as disease and phenotype, frequency, and genomic features that co-locate with a SVC;
- a simplified display in the Sequence Viewer as an aggregated histogram or density track from all studies (currently dbVar display each study as a track, which can be slow to render and difficult to display on small screens); and
- the ability to measure SSV concordance regions and validate across studies.

The Structural Variation Cluster project aimed to accomplish a number of goals. First, we generated a Genome Variant Format (GVF) file of SVC regions as defined above, based on RefSeq GRCh38 ^1^. Each region is assigned a unique ID (SVC1, SVC2, etc.). The SVC VCF file is used as the basis for generating aggregated data, filtering, generating sequence viewer tracks, and for comparison with user data. We also generated a histogram track to show the frequency of the regions across studies in genomic context for the Sequence Viewer. In addition, we annotated SVC regions with Gene, colocated dbSNP ^6^ reference SNPs, ClinVar ^7^, and other colocated features. We aimed to create a tool for filtering SVC GVFs by variant type, region size, region count, chromosome, and additional user-defined splitting and filtering parameters. This tool would allow users to compare their data with SVC GVFs and report matching regions of overlap.

## Methods

SVCs are defined as the union set of overlapping and non-overlapping regions for all SSVs aligned to the genome using HTSeq version 0.6.0 ^8^, based on the genomic coordinates in RefSeq human genome assembly GRCh38 (RefSeq accession GCF_000001405.26) ^1^ ( Figure 1).

### Structural variant cluster (SVC) from SSV

**Figure 2.**
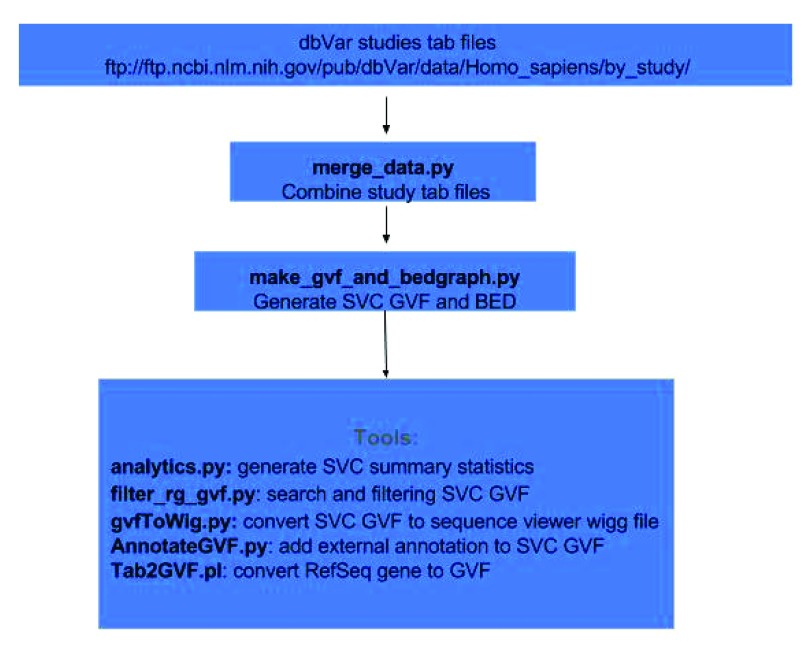
Dataflow for generating SVC and tools for analysis.

Figure 2 demonstrates the workflow for this analysis. dbVar SSV data by studies were obtained in tab delimited format from the FTPsite (ftp:// ftp.ncbi.nlm.nih.gov/pub/dbVar/data/Homo_sapiens/by_study/) and used as input. The study files were combined and sorted by chromosome positions into a single file using the script merge_data.py. SVC regions, including counts as shown in Figure 1, were generated from the merged file using the script make_gvf_and_bedgraph.py, which output SVC GVF and BED files. Since the approach in Figure 1 is similar to finding consensus regions or overlapping features between aligned reads make_gvf_and_bedgraph.py use HTSeq.GenomicInterval class to store SSV chr. start, and stop coordinates as genomic features and the HTSeq.GenomicArrayOfSets class to identify overlapping positions to generate SVC and counts.

Additional tools are available as scripts using SVC GVF as input to compute summary statistics, to search and filter, to generate WIG files for viewing in sequence viewer, and to annotate using external data sources. All scripts and examples are available on GitHub ( https://github.com/NCBI-Hackathons/Structural_Variant_Comparison/). For this study all coordinates reported are based on GRCh38.

## Results

### Computing structural variant cluster (SVC)

As shown in Figure 1, SVCs were created from overlapping and non-overlapping regions of two or more SSVs using the HTSeq.GenomicArrayOfSets class and output as GVF file format. Each SVC is counted for the number of times it is present as a subregion of a SSV, providing a total SVC count across studies. A single SSV by itself without any overlap between itself and another SSV in the region constitutes a single SVC with a feature count of 1. 3.6 million dbVar SSVs generated 3.4 million SVCs for all dbVar data (combined-set) by variant type ( Table 1).

**Table 1.** SVC count and percentage of total by variant types from combined-set across all studies. The most common variant type was deletion followed by CNV. All CNV types combined (rows 1, 7, and 8 in Table 1) total 972,335. We also generated SVC for each variant type (ie. CNV, in/del, etc.) and by individual studies (study-set) for QA/QC and analysis between types and studies of interest. The study-set generated a total of 2.5 million SVCs versus 3.4 million SVCs from the combined-set.

Variant Type	Percent Total (%)	SVC Count
deletion	35%	1194270
copy number loss	18%	633268
mobile element insertion	15%	517506
duplication	9%	303790
insertion	7%	232552
indel	5%	186658
copy number gain	5%	169876
copy number variation	5%	169191
Others	1%	17810
Total	100%	3424921

### Comparison of combined-set and study-set SVC derived from variant type CNV with ClinVar and genomic annotations

**Figure 3.**
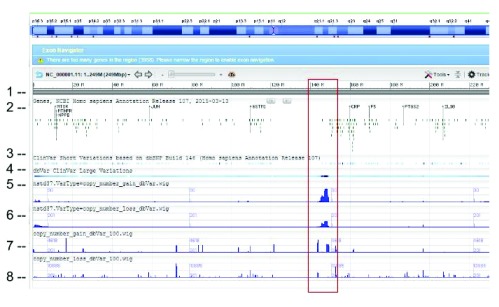
SVC (type=CNV) distribution on chromosome 1 as seen on NCBI Variation Viewer (Variation Viewer - NCBI). Starting from the top: (1) chr 1 sequence, (2) Gene track, (3) ClinVar short variation for dbSNP SNV, (4) ClinVar large variation, (5) ClinGen SVC study-set (dbVar:nstd37) copy number gain, (6) ClinGen SVC study-set (dbVar:nstd37) copy number loss, (7) SVC combined-set for copy number gain with count >= 100, and (8) SVC combined-set for copy number loss with count >= 100. The red box highlights an SVC hotspot region found in ClinGen (dbVar:nstd37) tracks 5 and 6 that correspond with the variants in ClinVar. The scale for SVC count histogram are 1–90 (track 5), 1–20 (track 6), 1–4618 (track 7), and 1–10885 (track 8).

**Figure 4.**
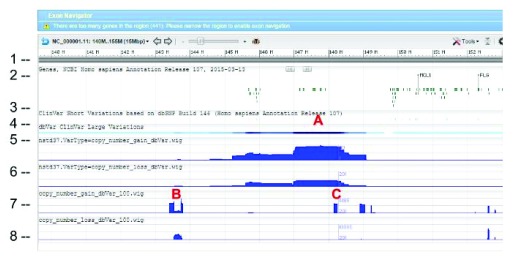
Zoomed in view of the red box region in Figure 1 showing dbVar:nstd37 SVC peak A corresponding to ClinVar variants and flanking peaks B and C from the common combined-set. The track and histogram scales are as described in Figure 5.

**Figure 5.**
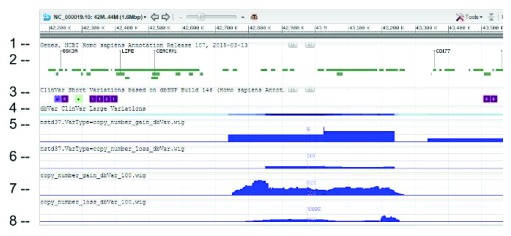
Magnified region of Chr19 showing a region of high density of variants where SVCs presumed to be common are also found. The tracks and histogram scales are as described in Figure 3.

WIG files were generated from SVC GVF files to allow loading into sequence viewer for quick visual inspection as shown in Figure 3. The SVC sets used for inspections are the combined-set which includes 1000Genomes ^9^, as well as other large studies to provide frequently occurring or “common” SVC to compare with presumed curated variants that have clinical significance from study-set (dbVar:nstd37) submitted by ClinGen ^10^. The Variation Viewer ^11^ allows for quick navigation by genes, chromosome positions, and variations for visual comparison ( Figure 3, Figure 4, and Figure 5). Figure 3 and Figure 4 show a hotspot peak A in ClinVar (track 4) that corresponds with a peak in SVC from nstd37, suggesting that this region is critical for function and that variations in this region are rare. These conclusions are supported by the lack of corresponding SVC peaks in the combined-set “common” tracks 7 and 8. However, tracks 7 and 8 also contain peaks B and C that flank the ClinVar peak, which may demarcate the boundaries for the critical region peak A. In contrast, Figure 5 shows that there are corresponding SVC peaks in the nstd37 (rare) and in the combined-set (common), suggesting that variants in this region may have minimal or no clinical impact by themselves.

## Conclusions

The software tools we developed and provide here compute SVCs and provide counts of concordance regions across SSVs. We also developed tools to search, filter, annotate, and graphically view the results in sequence viewers or to incorporate them into custom analysis pipelines. Using these tools, we provide examples ( Figure 3) for comparing across different SVC data sets with other annotation (such as genes and ClinVar). Such comparisons will allow users to investigate across the genome - or near a gene of interest - and to look for concordance and conflicts between data, which may help users form hypotheses regarding the biological impact of observed variation in SVC regions. In future, we will conduct the work and analysis required for SVC data quality assurance. We believe that SVC data promise to improve the analysis and the elucidation of the biological impact of structural variants, and in future, will probably have uses beyond those described here. Potential uses for SVC data could include:

- the evaluation of other SVC hot spot regions to determine if they occur biologically or are due to genome problem regions;
- the use of study metadata to validate SVCs that are in concordance with regions across studies and different assay platforms;
- the validation of rare SVCs (count =< 2) and common SVCs (count > 2);
- identification of evidence of variations in all public SRA data;
- combined analysis and annotation of SVCs to ClinVar, dbSNP, and other variation resources;
- the creation of a reference dbVar “SV” number based on SVCs, which would be the equivalent to dbSNP’s RS number;
- identification of population-specific SVCs to gain insight into the functional significance of structural variants and their evolution; and
- determination of high-priority SVCs with significant functional impact and effects.

In addition, a “dbVar Beacon Service” could be developed to allow users to query dbVar if variants exists for a genomic location of interest using combined SVC data. The results would report the number of SVCs and associated SSV IDs and study IDs. Users could then download the study or SSV of interest from dbVar.

## Software availability

Latest source code: https://github.com/NCBI-Hackathons/Structural_Variant_Comparison/

Archived source code as at time of publication: http://dx.doi.org/10.5281/zenodo.48201 ^12^

Accompanying wiki: https://github.com/NCBI-Hackathons/Structural_Variant_Comparison/wiki

Manual: https://docs.google.com/document/d/1WBnEnShnw28ZFg17A3xUpWOyvxXjb2q-h1kF-XYVWEw/edit?usp=sharing

License: CC0 1.0 Universal

## References

[1] Saeed S Bonnefond A Manzoor J: Genetic variants in *LEP*, *LEPR*, and *MC4R* explain 30% of severe obesity in children from a consanguineous population. *Obesity (Silver Spring).* 2015;23(8):1687–95. 10.1002/oby.21142 26179253

[2] Ross JS Badve S Wang K: Genomic profiling of advanced-stage, metaplastic breast carcinoma by next-generation sequencing reveals frequent, targetable genomic abnormalities and potential new treatment options. *Arch Pathol Lab Med.* 2015;139(5):642–9. 10.5858/arpa.2014-0200-OA 25927147

[3] Radke DW Lee C: Adaptive potential of genomic structural variation in human and mammalian evolution. *Brief Funct Genomics.* 2015;14(5):358–68. 10.1093/bfgp/elv019 26003631 PMC6278953

[4] Home - dbVar - NCBI [Internet]: Home - dbVar - NCBI.[cited 2016 Feb 24]. Reference Source

[5] Lappalainen I Lopez J Skipper L: DbVar and DGVa: public archives for genomic structural variation. *Nucleic Acids Res.* 2013;41(Database issue):D936–41. 10.1093/nar/gks1213 23193291 PMC3531204

[6] Sherry ST Ward MH Kholodov M: dbSNP: the NCBI database of genetic variation. *Nucleic Acids Res.* 2001;29(1):308–11. 10.1093/nar/29.1.308 11125122 PMC29783

[7] Landrum MJ Lee JM Benson M: ClinVar: public archive of interpretations of clinically relevant variants. *Nucleic Acids Res.* 2016;44(D1):D862–8. 10.1093/nar/gkv1222 26582918 PMC4702865

[8] Anders S Pyl PT Huber W: HTSeq--a Python framework to work with high-throughput sequencing data. *Bioinformatics.* 2015;31(2):166–9. 10.1093/bioinformatics/btu638 25260700 PMC4287950

[9] estd214 - 1000 Genomes Consortium Phase 3 - dbVar Study - NCBI [Internet]: estd214 - 1000 Genomes Consortium Phase 3 - dbVar Study - NCBI.[cited 2016 Feb 24]. Reference Source

[10] ClinGen - ClinGen Clinical Genome Resource [Internet]: ClinGen - ClinGen Clinical Genome Resource.[cited 2016 Feb 24]. Reference Source

[11] Variation Viewer - NCBI [Internet]: Variation Viewer - NCBI.[cited 2016 Feb 24]. Reference Source

[12] John G TriLe965 Hsu J: Structural_Variant_Comparison: Initial Post-Hackathon Release. *Zenodo.* 2016 Data Source

