# Supplementary material for: dbVar structural variant cluster set for data analysis and variant comparison
Source: F1000Res. 2017 Feb 28;5:673. Originally published 2016 Apr 13. [Version 2] doi: 10.12688/f1000research.8290.2 (PMC5345777; doi:10.12688/f1000research.8290.2)
Supplement: Peer review history

# Peer review: Referee response for version 2

**DOI:** 10.5256/f1000research.10614.r20693

**Published:** 2017-03-06

**Authors:** Lihua Julie Zhu

**Affiliations:** 1 Department of Molecular, Cell and Cancer Biology, Program in Bioinformatics and Integrative Biology, University of Massachusetts Medical School, Worcester, MA, USA

Thanks for addressing my comments!

I have read this submission. I believe that I have an appropriate level of expertise to confirm that it is of an acceptable scientific standard.

# Peer review: Referee response for version 1

**DOI:** 10.5256/f1000research.8916.r15157

**Published:** 2016-08-09

**Authors:** Justin Zook

**Affiliations:** 1 Genome-scale Measurements Group, National Institute of Standards and Technology, Gaithersburg, USA

The authors create a set of scripts that take SVs submitted to dbVar and find how many calls cover each region of the genome.  I expect these will be useful for understanding locations in the genome where multiple SV calls have been made.  The methods appear to be straightforward to use, so that they can be applied to new callsets as they are submitted to dbVar and potentially to other repositories as well. I have a few minor suggestions below:

1. Fig 4 caption seems to refer to red box in Fig 3, not Fig 1.
2. It appears that the output wig files for the current dbvar are on the GitHub site, and it would be useful to make clear that these are available in the paper.  Are the output bed files also available? Are the outputs available as a track in any NCBI browser?
3. Why did the authors choose gvf as the output format?  Although no format is great for SVs, would the authors consider adding vcf as an output format since vcf seems to be increasingly adopted by SV callers?
4. This is implied in the future work proposed, but it may be useful to state explicitly that dbVar entries are not curated for accuracy, so regions with many SVs may be enriched for artifacts or true SVs or both.

I have read this submission. I believe that I have an appropriate level of expertise to confirm that it is of an acceptable scientific standard.

# Peer review: Referee response for version 1

**DOI:** 10.5256/f1000research.8916.r13373

**Published:** 2016-05-05

**Authors:** Lihua Julie Zhu

**Affiliations:** 1 Department of Molecular, Cell and Cancer Biology, Program in Bioinformatics and Integrative Biology, University of Massachusetts Medical School, Worcester, MA, USA

dbVar is a database hosted by NCBI for archiving all types of genomic structural variants (GSV) in all species, including copy number variations (CNV), insertions, deletions, inversions, translocations, and complex chromosomal rearrangement. It accepts data submissions from researchers and exchanges data on a regular basis with the European Database of Genomic Variants Archive (DGVa). To facilitate the exchange, annotation, computation, visualization, reporting and interpretation of user submitted structural variants (SSVs), overlapping SSVs are merged to form a non-redundant set of genomic regions called structural variant clusters (SVCs), and utilities have been developed for annotating, searching, summarizing, filtering and visualizing SVC data in GVF format. However, in light of the previous publication of the same database (Lappalainen *et al.*, 2013), it is unclear to the reviewers about the additional contribution of this manuscript. It would be important if the authors could cite the previous publication and clearly describe detailed updates made to the database and how these updates improve the existing software to help reviewers to understand what is new.

It would be helpful if the authors could clarify the reason why Table 1 does not add up to 100%, and describe where 2.5 million SVCs are derived from as stated as "The study-set generated a total of 2.5 million SVCs versus 3.4 million SVCs from the combined-set ". In addition, the resolution for Figure 2-5  needs to be improved.

I have read this submission. I believe that I have an appropriate level of expertise to confirm that it is of an acceptable scientific standard, however I have significant reservations, as outlined above.

## References

[1] LappalainenILopezJSkipperLHefferonTSpaldingJDGarnerJChenCMaguireMCorbettMZhouGPaschallJAnanievVFlicekPChurchDM: DbVar and DGVa: public archives for genomic structural variation. *Nucleic Acids Res*.2013;41(Database issue) : 10.1093/nar/gks1213 D936-41 10.1093/nar/gks1213 23193291PMC3531204
